# Influence of tibial slope asymmetry on femoral rotation in patients with lateral patellar instability

**DOI:** 10.1007/s00167-012-2247-4

**Published:** 2012-10-25

**Authors:** Peter Balcarek, Annika Terwey, Klaus Jung, Tim Alexander Walde, Stephan Frosch, Jan Philipp Schüttrumpf, Martin Michael Wachowski, Henning Dathe, Klaus Michael Stürmer

**Affiliations:** 1 Department of Trauma Surgery, Plastic and Reconstructive Surgery, University Medicine Göttingen, Robert-Koch-Str. 40, 37075 Göttingen, Germany; 2 Medical Statistics, University Medicine Göttingen, Göttingen, Germany; 3 AG Biomechanics, Orthodontics, University Medicine Göttingen, Göttingen, Germany

**Keywords:** Patellar instability, Tibial slope, Femoral rotation, Biomechanics

## Abstract

**Purpose:**

The geometry of the tibial plateau and its influence on the biomechanics of the tibiofemoral joint has gained increased significance. However, no quantitative data are available regarding the inclination of the medial and lateral tibial slope in patients with patellar instability. It was therefore the purpose of this study to evaluate tibial slope characteristics in patients with patellar dislocations and to assess the biomechanical effect of medial-to-lateral tibial slope asymmetry on lateral patellar instability.

**Methods:**

Medial and lateral tibial slope was measured on knee magnetic resonance images in 107 patients and in 83 controls. The medial-to-lateral tibial slope asymmetry was assessed as the intra-individual difference between the medial and lateral tibial plateau inclination considering severity of trochlear dysplasia. The effect of tibial slope asymmetry on femoral rotation was calculated by means of radian measure.

**Results:**

Severity of trochlear dysplasia was significantly associated with an asymmetric inclination of the tibial plateau. Whereas the medial tibial slope showed identical values between controls and study patients (n.s.), lateral tibial plateau inclination becomes flatter with increasing severity of trochlear dysplasia (*p* < 0.01). Consequently, the intra-individual tibial slope asymmetry increased steadily (*p* < 0.01) and increased internal femoral rotation in 20° and 90° of knee flexion angles in patients with severe trochlear dysplasia (*p* < 0.01). In addition, the extreme values of internal femoral rotation were more pronounced in patients with patellar instability, whereas the extreme values of external femoral rotation were more pronounced in control subjects (*p* = 0.024).

**Conclusion:**

Data of this study indicate an association between tibial plateau configuration and internal femoral rotation in patients with lateral patellar instability and underlying trochlear dysplasia. Thereby, medial-to-lateral tibial slope asymmetry increased internal femoral rotation during knee flexion and therefore might aggravate the effect of femoral antetorsion in patients with patellar instability.

**Level of evidence:**

III.

## Introduction

Lateral patellar dislocation (LPD) is subject to a constant advancement of biomechanical understanding and treatment modalities. In recent years, it has become obvious that numerous risk factors have to be considered in clinical decision-making and that the understanding of an individual’s anatomy is essential to identify who is at risk for subsequent instability episodes and to choose the most appropriate treatment [15].

Patellar stability is maintained by a complex interplay of active, passive and static stabilizers that act in harmony during knee motion [15]. The static stabilizers are composed mainly of the joint geometry; particularly, the shape of the trochlear groove prevents LPD, predominantly at ranges between 20° and 30° of knee flexion [16]. Concomitantly, abnormal joint geometry influences patellofemoral instability by means of different grades of trochlear dysplasia, by the anatomical and mechanical axis of the femur and tibia (genu valgum), increased femoral antetorsion and external tibial rotation, patella height, and by an increased tibial tuberosity–trochlear groove distance (TT–TG) [3].

In recent years, the geometry of the tibial plateau and its influence on the biomechanics of the tibiofemoral joint has gained increased significance. Numerous investigators have evaluated the effect of tibial slope inclination on tibiofemoral contact area, joint translation, rotation and on the strain biomechanics of the cruciate ligaments [6, 7, 15]. In addition, knee anatomy, particularly the tibial slope, is directly associated with knee biomechanics exhibited during dynamic landings [9]. Although, the importance of the inclination of the tibial plateau is well established in the current literature, no quantitative data are available regarding the inclination of the medial and lateral tibial plateau and its influence on the biomechanics of the tibiofemoral joint in patients with LPD. Therefore, it was the objective of this study to evaluate the geometry of the tibial plateau in patients with LPD. Specifically, we addressed the following research questions: (a) Is lateral patellar instability associated with a modified inclination of the medial and lateral tibial plateau (tibial slope) and, if so, (b) in what way does this modification influence tibiofemoral biomechanics? It was the hypothesis of the current study that patients with patellofemoral instability show also significant changes in the anatomy and the biomechanics of the tibiofemoral joint.

## Materials and methods

Patient selection was performed according to Balcarek et al. [2]. The study group was composed of 107 patients (male/female, 55/52; age range 10–55 years) with 107 knee magnetic resonance imaging (MRI) investigations who had been treated for LPD between February 2006 and June 2010. A picture archiving and communications system (PACS) workstation (Centricity, GE Healthcare, St. Gilles, United Kingdom) was used to identify patients with acute or recurrent patellar dislocations. The criteria for LPD included joint effusion, contusion on the lateral femoral condyle or medial patella margin, osteochondral fragments, injury to the medial ligamentous stabilizers and a lateralized patella. The MRI-based diagnosis of LPD was made in patients who met three or more of the criteria. Additionally, to be included patients had to have a convincing history of primary or recurrent patellar dislocations with the clinical symptom of giving way and the clinical findings of joint effusion, tenderness along the medial retinaculum, medial patella or medial femoral condyle, and a positive apprehension sign.

The criteria for exclusion were any proximal or distal realignment procedures, trochleoplasty, fractures of the distal femur or tibial head, and a multiligament-injured knee joint. Standard knee arthroscopy with medial reefing was not an exclusion criterion. These criteria were used to identify 107 patients, who formed the study group for this investigation.

## Control group

Knee MRI investigations of 83 patients (male/female, 42/41; age range 12–59 years) served as controls. Investigations were performed during the same time period, and patients were matched for age and gender. Investigations were performed due to an internal derangement of the knee such as meniscus tear or cartilage lesion of the tibiofemoral joint. Patients with osteoarthritis or other objective pathologies related to the patellofemoral joint were excluded. In addition, patients with an anterior cruciate ligament (ACL) tear were also excluded because this injury is often associated with an increased inclination of the tibial plateau in non-contact ACL injury mechanisms [6].

## Image evaluation

In all patients coronal, sagittal and transverse MRI images were available and were used to measure the medial and lateral tibial slope, the anatomical distal lateral femur angle (aDLFA), the mechanical proximal medial tibial angle (mPMTA), and the degree of trochlear dysplasia. Measurements were performed with the use of the annotation tools on the digital PACS workstation. The software presented the angular values and length measurements to one decimal place that were rounded into a single-digit integer format. This means, if the true value of an angle was, for example, 5.4°, this was rounded off to 5°. On the other hand, if the actual angle was 5.5°, this was rounded up to 6°. Similarly, if the measured length was 40.5 mm, this was rounded up to 41 mm, and if the actual length was 40.4 mm, this was rounded off to 40 mm. Therefore, all measurements were made with a sensitivity of 1° (−0.5° to 0.5°) and 1 mm (−0.5 to 0.5 mm), respectively [5].

**Fig. 1:**
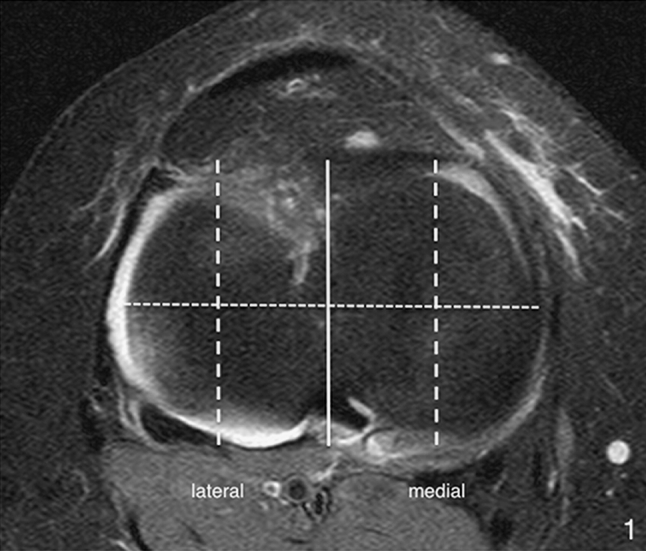
First transverse MR image in craniocaudal direction that shows the entire tibial head. In this transverse image, the corresponding sagittal slices located most closely to the tibial head centre (*solid line*) and in the centre of the medial and lateral tibial plateau (*dashed lines*) were determined. Similarly, the corresponding coronal section located most closely to the tibial head centre (*dotted line*) was established

**Fig. 2:**
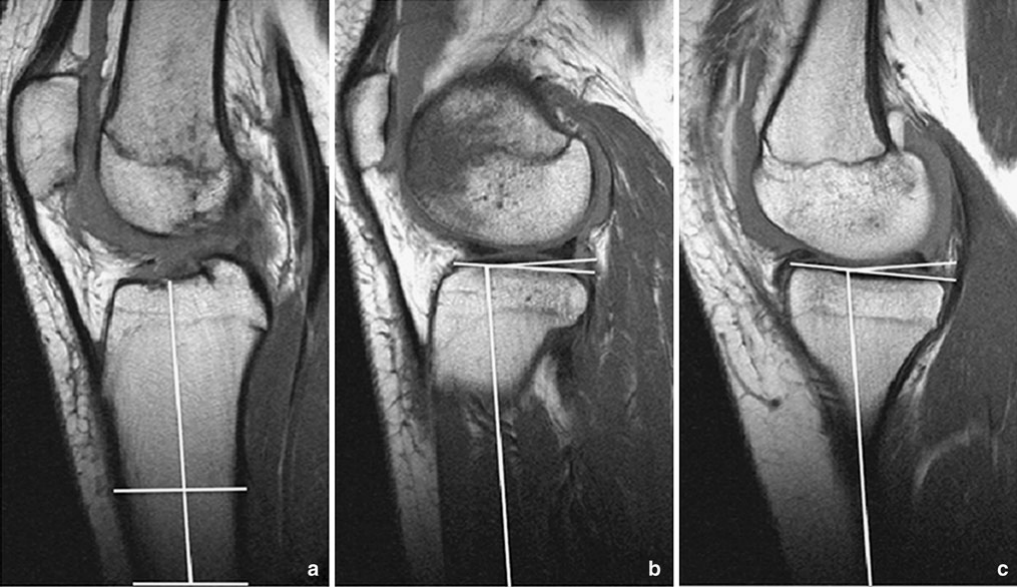
The sagittal plane that represents the corresponding image to the *solid line* in Fig. 1 is shown in (**a**). This image was used to identify the longitudinal axis of the tibial diaphysis. The longitudinal axis was defined as the *midpoint* of the anterior–posterior width of the tibia at two points located 4–5 cm distally to the *joint line* and as distally as possible. To measure the medial and lateral tibial slope, the longitudinal axis was assigned to the corresponding planes in the centre of the lateral tibial plateau (**b**) and in the centre of the medial tibia plateau (**c**) as shown as *dashed lines* in Fig. 1. The conjugation line between the peak anterior and posterior points of the tibia plateau measured the inclination of the tibial slope perpendicular to the longitudinal axis of the tibia

To answer the first research question, the medial and lateral tibial slope was assessed according to the protocol proposed by Hashemi et al. [5]. Initially, the first proximal slice on transverse MR images that showed the entire tibial head was identified (Fig. 1). In this transverse image, the corresponding sagittal slice located most closely to the tibial head centre was determined (solid line in Fig. 1). This sagittal plane, which is shown in Fig. 2a, was then used to identify the longitudinal axis of the tibial diaphysis. The longitudinal axis was defined as the midpoint of the anterior–posterior width of the tibia at two points located 4–5 cm distally to the joint line and as distally as possible. The conjugation line of these two points represents the longitudinal axis of the tibia in the sagittal plane. To measure the medial and lateral tibial slope, the longitudinal axis was assigned to the corresponding planes in the centre of the medial tibial plateau and in the centre of the lateral tibia plateau, shown as dashed lines in Fig. 1. Again, the corresponding planes were reproduced in the medial and lateral sagittal image (Fig. 2b, c). The conjugation line between the peak anterior and posterior points of the bony tibia plateau measured the inclination of the tibial slope perpendicular to the longitudinal axis of the tibia. The tibial slope was defined as positive if the peak anterior point lay above the peak posterior point and was defined as negative if the posterior point lay above the anterior point.

**Fig. 3:**
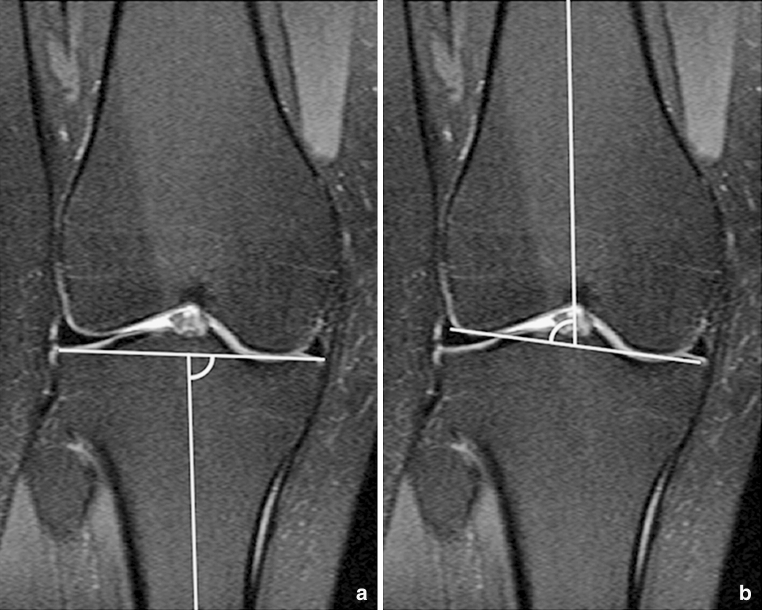
The longitudinal axis of the femur and the tibia was established in the frontal plane referencing most closely to the centre of the tibial head in the corresponding transverse image (*dotted line* in Fig. 1). The longitudinal axis was defined as the midpoint of the medial-to-lateral width of the tibia as distally as possible and at the midpoint of the tibial head. A similar approach was used to establish the diaphyseal axis of the femur in the coronal plane. The aDLFA and mPMTA was then measured as the angle between the longitudinal axis of the femur and tibia and the joint line represented by the most distally located points of the femoral condyles, and the peak points of the medial and lateral tibial plateau. *aDLFA* anatomical distal lateral femur angle, *mPMTA* mechanical proximal medial tibial angle

A comparable method was used to measure the aDLFA and mPMTA, according to Paley et al. [13]. First, the longitudinal axis of the tibia in the frontal plane was established referencing most closely to the centre of the tibial head in the corresponding transverse image (dotted line in Fig. 1). Again, the longitudinal axis was defined as the midpoint of the medial-to-lateral width of the tibia as distally as possible and at the midpoint of the tibial head. A similar approach was used to establish the diaphyseal axis of the femur in the coronal plane. The aDLFA and mPMTA were then measured as the angle between the longitudinal axis of the femur and tibia and the joint line represented by the most distally located points of the femoral condyles, and the peak points of the medial and lateral tibial plateau, respectively (Fig. 3a, b).

Trochlear dysplasia was assessed on the transverse MR images as described by Fucentese et al. [4]. The classification system was performed according to Dejour et al. [3]: Type A: Trochlear morphology preserved with a fairly shallow trochlea; Type B: Flat or convex trochlea; Type C: Asymmetry of trochlear facets with convex lateral facet and hypoplastic medial facet; and Type D: Asymmetry of trochlear facets, hypoplastic medial facet and cliff pattern.

**Fig. 4:**
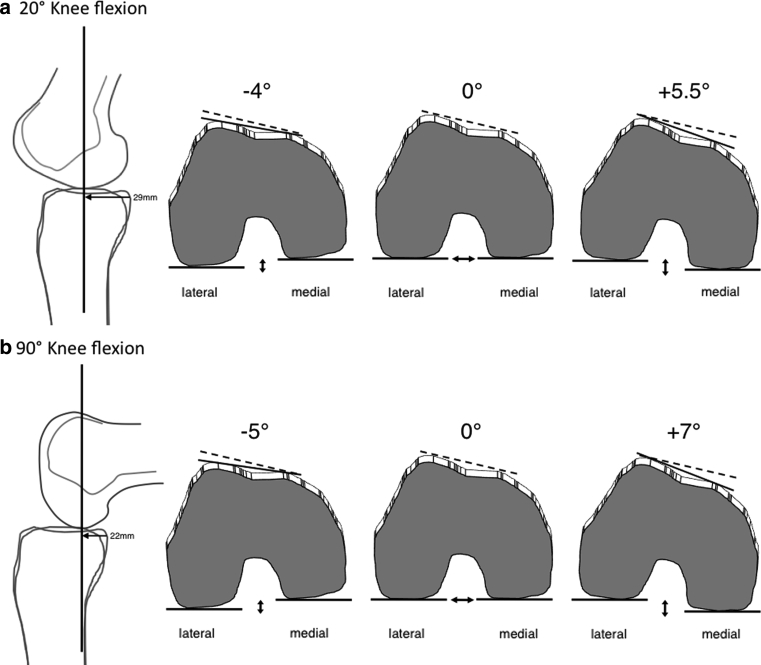
Illustrated is the effect of tibial slope asymmetry on femoral rotation by means of a difference in height between the medial and lateral tibial plateau in 20° and 90° of knee flexion. In the *frontal view*, the maximal observed effect on internal and on external femoral rotation is shown in comparison with the neutral position of the femoral condyles in both knee flexion angles

To answer the second research question, the analyses by Nägerl et al. [11] and Pinskerova et al. [14] were taken into account. These studies showed that the contact areas of the femoral condyles on the tibial plateau move backwards during knee flexion. Under weight-bearing conditions, the contact point between the medial femoral condyle and the tibial plateau averaged a distance of 29 and 22 mm measured from the ipsilateral posterior tibial cortex in 20° and 90° of knee flexion, respectively. This means that a difference of the medial and lateral tibial plateau inclination would influence femoral rotation by means of a different height between the posteromedial and posterolateral femorotibial contact points as illustrated in Fig. 4a and b. Thus, the medial-to-lateral tibial slope asymmetry was assessed as the intra-individual difference between the medial and lateral tibial plateau inclination. This means that the tibial slope asymmetry had a positive sign in cases where the medial slope was steeper than the lateral slope, and had a negative sign in cases where the medial slope was flatter than the lateral slope.

The effect of tibial slope asymmetry on femoral rotation was calculated mathematically by means of radian measure with the knee positioned in 20° and 90° of knee flexion. First, the value of tibial slope asymmetry (in degrees) was converted into radian according to the formula rad = deg π/180. This value was then multiplied either by 23 mm or by 30 mm, representing the difference in height (in mm) between the posteromedial and posterolateral contact points in 20° or 90° knee flexion. The value of 23 and 30 mm resulted from the length of the tibial head in the sagittal plane (Ø 52 mm; *n* = 35) minus the distance from the posterior tibial cortex to the medial femorotibial contact point (29 mm for 20° of knee flexion; 22 mm for 90° of knee flexion), according to Pinskerova et al. [14]. Subsequently, the differences in height were divided by the distance between the centre of the medial and lateral femoral condyle in the frontal plane (Intercondylar distance: Ø 44 mm; *n* = 35) representing the value of femoral rotation in radian. Values of radian measures were then converted into degrees (deg = rad 180/π) representing internal femoral rotation in cases of positive values and external femoral rotation in cases of negative values.

### Statistical analysis

Data are presented as frequencies, mean ± standard deviation and range. The influence of the grade of trochlear dysplasia onto the medial and lateral tibial slope and onto age, aDLFA, and mPMTA was individually assessed by linear regression with trochlear dysplasia as independent variable. Since age was significantly associated with the grade of trochlear dysplasia, it was added as an independent variable into the other regression models. Gender and LPD were compared between the different types of trochlear dysplasia using the χ²-exact test. The portion of study patients and controls on the 20 largest slope asymmetries, in positive and in negative direction, respectively, was evaluated by Fisher’s exact test. Because LPD and trochlear dysplasia were nearly perfectly correlated, an adjustment for age was not possible for this analysis.

To study intrarater and interrater reliability, two measurement series on 20 randomly taken MRI were drawn either repeatedly by 1 single rater with a 2-week interval or independently by 2 different raters. Reliability was assessed by the correlation (Pearson r) between the two measurement series or by the mean difference (*t* test) between them. All analyses were performed using the free software R (version 2.12, www.r-project.org). The testing level was chosen to be alpha = 5 % for all tests.

## Results

**Fig. 5:**
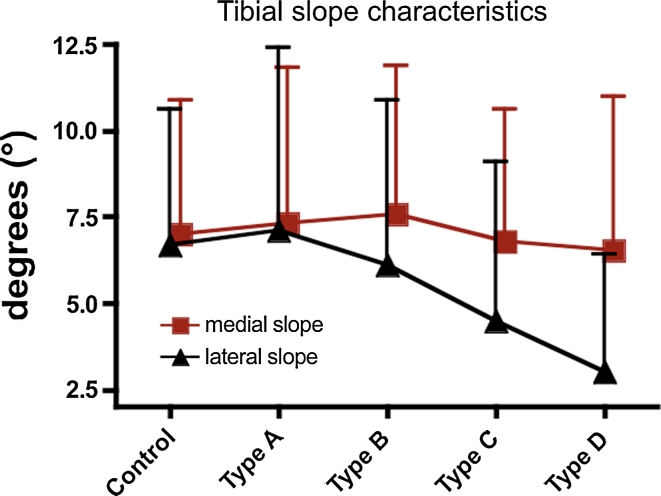
Shown are the mean values and SD of the medial and the lateral tibial slope in controls and in patients considering different grades of trochlear dysplasia

**Fig. 6:**
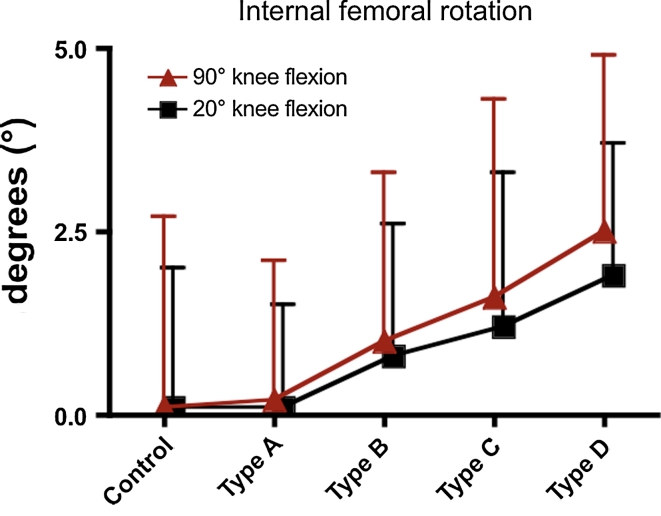
Shown are the mean values and SD of internal femoral rotation in controls and in patients considering different grades of trochlear dysplasia with the knee positioned in 20° and 90° of knee flexion

Severity of trochlear dysplasia was significantly associated with an asymmetric inclination of the tibial plateau (Table 1). Whereas the medial tibial slope showed identical values between controls and study patients (n.s.), lateral tibial plateau inclination becomes flatter with increasing severity of trochlear dysplasia (*p* < 0.01) (Table 1; Fig. 5). Consequently, the intra-individual slope asymmetry increased steadily towards positive values (*p* < 0.01) and therefore increased internal femoral rotation in 20° and in 90° of knee flexion in patients with severe trochlear dysplasia (*p* < 0.01) (Table 1; Fig. 6). Though tibial slope asymmetry showed a relatively wide range in both study and control patients, study patients were more frequently found than controls among the 20 largest positive values of tibial slope asymmetry (6°–10°). On the other hand, among the 20 largest negative values of tibial slope asymmetry (−4° to −8°), control patients were more often found than study patients (*p* = 0.024). Concomitantly, this means that the extreme values of internal femoral rotation were more pronounced in patients with LPD, whereas the extreme values of external femoral rotation were more pronounced in control subjects. The study group also showed a slightly increased valgus deformity expressed by a smaller aDLFA (*p* < 0.01), whereas the mPMTA did not differ between study and control subjects (n.s.). Interrater and intrarater reliability was near-perfect for the investigated parameters with a clearly positively significant correlation between the two measurement series (Table 2a, b).

**Table 1:** Distribution of patient characteristics and study parameters considering different grades of trochlear dysplasia

Parameter	Grade of trochlear dysplasia					*p* value
	Normal (*n* = 90)	Type A (*n* = 18)	Type B (*n* = 47)	Type C (*n* = 23)	Type D (*n* = 12)	
Age (years)	27.2 ± 9.6 (12.0–59.0)	22.6 ± 7.1 (13.0–38.0)	24.9 ± 9.7 (10.0–55.0)	20.5 ± 5.3 (13.0–30.0)	18.0 ± 6.7 (12.0–38.0)	<0.01
Gender						n.s.
Male	48 (53 %)	9 (50 %)	26 (55 %)	10 (43 %)	5 (42 %)	
Female	42 (47 %)	9 (50 %)	21 (45 %)	13 (47 %)	7 (58 %)	
Dislocation						<0.01
Yes	7 (8 %)	18 (100 %)	47 (100 %)	23 (100 %)	12 (100 %)	
No	83 (92 %)	0 (0 %)	0 (0 %)	0 (0 %)	0 (0 %)	
Tibial slope medial	7.0 ± 3.9 (−1.0 to 15.0)	7.3 ± 4.5 (0.0–14.0)	7.6 ± 4.3 (−1.5 to 20.0)	6.8 ± 3.8 (−0.5 to 14.0)	6.5 ± 4.5 (1.0–17.0)	n.s.*
Tibial slope lateral	6.7 ± 3.9 (−0.5 to 15.5)	7.1 ± 5.3 (0.0–15.5)	6.1 ± 4.8 (0.0–18.0)	4.5 ± 4.6 (−5.0 to 14.5)	3.0 ± 3.4 (−1.0 to 9.5)	<0.01*
Slope asymmetry (°)	0.2 ± 3.8 (−8.0 to 7.0)	0.2 ± 2.8 (−4.5 to 5.0)	1.5 ± 3.4 (−8.0 to 9.0)	2.3 ± 3.8 (−4.0 to 10.0)	3.5 ± 3.4 (−1.5 to 8.0)	<0.01*
Femoral rotation in 20° knee flexion	0.1 ± 1.9 (−4.2 to 3.7)	0.1 ± 1.4 (−2.4 to 2.6)	0.8 ± 1.8 (−4.2 to 4.7)	1.2 ± 2.1 (−2.3 to 5.5)	1.9 ± 1.8 (−0.7 to 4.2)	<0.01*
Femoral rotation in 90° knee flexion	0.1 ± 2.6 (−5.5 to 4.8)	0.2 ± 1.9 (−3.0 to 3.4)	1.0 ± 2.3 (−5.5 to 6.1)	1.6 ± 2.7 (−3.0 to 7.2)	2.5 ± 2.4 (−1.0 to 5.5)	<0.01*
aDLFA (°)	82.8 ± 2.2 (76.0–88.0)	81.3 ± 2.4 (76.5–86.0)	81.6 ± 2.4 (77.0–86.0)	81.0 ± 3.0 (76.0–86.5)	81.5 ± 2.4 (77.0–85.5)	<0.01*
mPMTA (°)	86.1 ± 2.5 (80.5–91.0)	85.3 ± 2.1 (82.0–88.5)	85.8 ± 2.2 (82.0–90.0)	86.0 ± 2.0 (82.0–90.0)	86.4 ± 2.1 (83.0–89.0)	n.s.*

Descriptive values are presented as either absolute (relative) frequencies or mean ± standard deviation (minimum–maximum). *p* values marked by an * are adjusted for age

*n.s.* Not significant, *aDLFA* anatomical distal lateral femur angle, *mPMTA* mechanical proximal medial tibial angle

**Table 2:** Correlation and mean of differences between 2 measurement series on the same 20 individuals, (a) drawn repeatedly by 1 single rater, (b) 2 different raters

	Pearson r	*p* value	Mean of differences	*p* value
a *Intrarater reliability*				
Medial slope	0.92	<0.01	0.4	n.s.
Lateral slope	0.95	<0.01	0.95	n.s.
b *Interrater reliability*				
Medial slope	0.85	<0.01	0.38	n.s.
Lateral slope	0.88	<0.01	0.91	n.s.

*n.s.* not significant

## Discussion

The most important finding of this study was that the individual tibial slope asymmetry, expressed as the difference between the medial and the lateral tibial slope, increased linearly considering severity of trochlear dysplasia. This increase in tibial slope asymmetry was based on a decrease in the lateral tibial slope with consistent inclination of the medial slope. Tibial slope asymmetry therefore influenced femoral rotation by means of a different height between the posterior medial and posterior lateral tibial head. Thereby, internal femoral rotation was more pronounced in patients with LPD, whereas external femoral rotation was more pronounced in control subjects without patellofemoral instability.

Links between knee joint biomechanics and joint morphology have been well described in recent years [1, 10]. Particularly, the effect of the geometry of the tibial plateau on the strain biomechanics of the cruciate ligaments has gained increased significance. Thereby, the combination of steep medial and lateral tibial slopes with a shallow concavity of the medial tibial head increased the risk of non-contact ACL injuries [6]. Moreover, the geometry of the tibial slope also influenced the peak stance knee internal rotation during dynamic landings [9]. Specifically, when the lateral tibial slope dominated tibial plateau geometry, the peak internal rotation angle was increased, indicating that the geometry of the tibial plateau exerts a dynamic role on knee rotational alignment. Similarly, our results support an association between tibial plateau configuration and internal femoral rotation in patients with lateral patellar instability and underlying trochlear dysplasia. Thereby, medial-to-lateral tibial slope asymmetry increased internal femoral rotation during knee flexion, potentially aggravating the effect of femoral antetorsion in patients with LPD. In addition, it seems plausible that tibial slope asymmetry also changes the knee axial rotation response [9]. With the longitudinal tibial rotation axis located on the medial tibial plateau [19], a relative increase in the medial slope will shift the medial tibiofemoral contact point anteriorly. Concomitantly, a relative shallow lateral slope will reduce the anterior directed tibiofemoral translation force on the lateral side, culminating in greater external tibial rotation. Thus, positive values of tibial slope asymmetry might influence patellofemoral instability by both, an increase in internal femoral rotation and an increase in external tibial rotation.

One has to consider that a smaller radius of curvature of the lateral femoral condyle might reduce the effect on internal femoral rotation. Three distinct features—the femoral bicondylar angle, the prominence of the lateral lip of the femoral trochlear, and the elliptic profile of the lateral condyle—characterize the development of the trochlear groove and the distal femur [18]. The increase in bicondylar angle is correlated with the disproportionate anteroposterior development of the lateral pillar. This in turn contributes to increasing the lateral condyle′s radius of curvature [17]. We therefore measured the radius curvature of 35 study patients (Trochlear dysplasia Type C and D) and of 35 at random selected patients of the control group. No significant difference was found between both groups (data not shown) so that the lateral condyle′s radius of curvature seems unlikely to compensate for the asymmetric tibial slope effect.

Recently, Hashemi et al. [5] have already pointed out that the true tibial slope should be measured at the centre of the articular regions of the medial and lateral tibial plateau. They found a relatively wide range for both the medial and the lateral slope with ranges between −3° and 10° and between 0° and 14°, respectively. Similarly, Matsuda et al. [8] reported medial and lateral tibial slope ranges between 5° and 15.5° and between 0° and 14.5°, respectively. We also found a high range within measurements of medial (−1.5° to 20°) and lateral tibial slope (−5° to 18°), moderately exceeding those of previous studies. However, this difference might be attributable to a much higher number of subjects included in our study (*n* = 190) when compared to the analysis by Hashemi et al. (*n* = 55) and Matsuda et al. (*n* = 60) [5, 8].

To the best of our knowledge, this is the first study that investigated the association between LPD and tibial plateau geometry. In addition, this study aims to provide an initial understanding of medial-to-lateral tibial slope asymmetry and the resultant biomechanical characteristics of the tibiofemoral joint with its relevant contributions to lateral patellar instability. If one considers the mean values of tibial slope asymmetry and femoral rotation, it could be argued that they may not have a dramatic effect on the biomechanics of the knee joint. However, the important point to consider is the differences between the extreme values of internal femoral rotation (5.5° in 20° knee flexion; 7.2° in 90° knee flexion) in patients with patellar instability and that of external femoral rotation (−4.2° in 20° knee flexion; −5.5° in 90° knee flexion) in control subjects. In addition, further limitations were noticed and deserve mention. First, as already stated by Hashemi et al. [5], it is important to note that the access to a sufficient length of the femur and tibia in the magnetic resonance image and the ability to identify landmarks, precisely all, could have an impact on the slope measurements. Second, slope measurements were referenced on the bony landmarks in the centre of the medial and lateral tibial plateau, but knee joint morphology is further characterized by the cartilage surface that is concave medially and convex laterally. This means that the articular surface is higher on the lateral side and lower on the medial side when compared to the bony landmarks used in this study. This in turn, however, would enhance the observed effect on internal femoral rotation during knee flexion rather than diminish it. Finally, our analyses were based on an idealized mathematical model with the knee positioned in 20° and 90° of knee flexion. It was not able to evaluate the effect of tibial slope asymmetry on tibiofemoral biomechanics during varying degrees of knee flexion. If one considers that the patella becomes least stable between 0° to 30° of knee flexion [16], it could be argued that an increase in internal femoral rotation may not have a dramatic effect on patellar instability in higher degrees of knee flexion. However, though most patellae dislocate from a nearly straight start followed by a movement to flexion, there is a certain amount of patients dislocating their patellae from a well-bent start followed by either knee extension or flexion [12]. Thus, it seems likely that an increase in internal femoral rotation contributes relevantly to patellar instability even in higher degrees of knee flexion.

Knowledge of the influence of medial-to-lateral tibia slope asymmetry on femoral rotation might help physicians in the decision-making process towards a torsional osteotomy in patients with lateral patellar instability and a marginally increased femur antetorsion.

## Conclusion

The present study introduces a new aspect in the complex interplay between the patellofemoral and the tibiofemoral joint considering the biomechanics of lateral patellar instability. It shows that the individual tibial slope asymmetry, expressed as the difference between the medial and the lateral tibial slope, increased linearly considering the severity of trochlear dysplasia and, concomitantly, influenced femoral rotation during knee flexion. Thereby, internal femoral rotation was more pronounced in patients with LPD, whereas external femoral rotation was more pronounced in control subjects without patellofemoral instability.
